# The effects of intrinsic motivation on mental fatigue

**DOI:** 10.1371/journal.pone.0243754

**Published:** 2021-01-04

**Authors:** Mega B. Herlambang, Fokie Cnossen, Niels A. Taatgen

**Affiliations:** 1 Bernoulli Institute for Mathematics, Computer Science and Artificial Intelligence, University of Groningen, Groningen, The Netherlands; 2 Department of Industrial Engineering, Institut Teknologi Indonesia, South Tangerang, Indonesia; University of Wuerzburg, GERMANY

## Abstract

There have been many studies attempting to disentangle the relation between motivation and mental fatigue. Mental fatigue occurs after performing a demanding task for a prolonged time, and many studies have suggested that motivation can counteract the negative effects of mental fatigue on task performance. To complicate matters, most mental fatigue studies looked exclusively at the effects of extrinsic motivation but not intrinsic motivation. Individuals are said to be extrinsically motivated when they perform a task to attain rewards and avoid punishments, while they are said to be intrinsically motivated when they do for the pleasure of doing the activity. To assess whether intrinsic motivation has similar effects as extrinsic motivation, we conducted an experiment using subjective, performance, and physiological measures (heart rate variability and pupillometry). In this experiment, 28 participants solved Sudoku puzzles on a computer for three hours, with a cat video playing in the corner of the screen. The experiment consisted of 14 blocks with two alternating conditions: low intrinsic motivation and high intrinsic motivation. The main results showed that irrespective of condition, participants reported becoming fatigued over time. They performed better, invested more mental effort physiologically, and were less distracted in high-level than in low-level motivation blocks. The results suggest that similarly to extrinsic motivation, time-on-task effects are modulated by the level of intrinsic motivation: With high intrinsic motivation, people can maintain their performance over time as they seem willing to invest more effort as time progresses than in low intrinsic motivation.

## 1 Introduction

Mental fatigue is a subjective feeling and a psychobiological condition after doing a demanding cognitive task for a long time [1–3]. Typically, when an individual is mentally fatigued, performance levels, e.g., in sustained attention and working memory tasks, decline [4–7], and the inclination to look for more rewarding activities increases [8–11]. In addition, prolonged mental fatigue impairs physical performance [2, 12, 13] and leads to safety issues, such as traffic accidents and errors in the workplace [14–16].

In general, there are two theories for explaining the effects of mental fatigue: declining resources and lack of motivation [5, 9]. The first theory suggests that finite resources and failure in allocating resources cause performance to decrease. Support for this theory is found in an experiment where doing a demanding task for a long time suppressed the brain activity [17]. Moreover, the resource theory has been the prime explanation for performance decrement especially in vigilance tasks [18].

In contrast, the second theory suggests that impaired performance is caused by amotivation, which is the lack of desire to continue doing an activity [8, 19]. In most cases, performing a cognitively demanding task for a long time increases the feeling of fatigue. As it increases, one will be less willing to stay engaged with the task, i.e., less motivated to continue performing the task [20]. As a result, a lower level of motivation impairs performance [10, 20]. However, prolonged tasks do not necessarily lower performance if the individual who performs the task is motivated: The individual is able to maintain or find another source of motivation. For instance, after doing a demanding task for a few hours, performance levels decreased but returned to the initial level when participants were offered external rewards at the end of the block [11]. A similar study by Boksem et al. [21], where they offered participants a monetary reward 20 min before the experiment ended, showed that performance increased significantly during this last block. In our own study, where we manipulated rewards continuously for 2.5 hr and asked participants to do a demanding working memory task to count and calculate the total number of vowels, showed that in reward blocks, and in contrast to nonreward blocks, participants were able to maintain performance, invested more mental effort (subjectively and physiologically by using heart rate variability), and were less distracted [9]. These results show a clear effect of motivation on mental fatigue, which are difficult to explain by the resource theory.

Motivation is different from, but related to, effort. Motivation refers to the “activating orientation of current life pursuits toward a positively evaluated goal state” [22 p. 15]. It drives an individual to perform a particular activity, and the individual may behave differently according to the level of motivation. Effort, on the other hand, refers to “the degree of engagement with demanding tasks” [23 p. 396] and reflects a feeling resulting from the cost/benefit calculation of doing a task that later determines performance [24]. For instance, when one feels fatigued, which corresponds to a high level of perceived effort [12, 13], one will choose to continue doing the task, maintaining performance, or let one’s performance level drop based on the result of the calculation [8, 24].

Furthermore, the effects of a highly motivated individual on his/her performance may be mediated by increased effort. A study by Gendolla and colleagues [25] showed that the level of effort correlated with the subjective difficulty of the task according to its feasibility and reasonability. More specifically, if performing a task is viewed as beneficial and viable, the more difficult the task is, the higher the effort will be [26]. Their notion came from the motivation intensity theory [27], which posits that human beings attempt to avoid using unnecessary resources and have what is called potential motivation, which is the level of motivation that determines how much effort an individual is willing to invest based on the difficulty, feasibility, and benefits of performing the task, which resembles the cost/benefit calculation [24].

With regard to the motivational theory of mental fatigue, broadly, there are two types of motivation: extrinsic and intrinsic motivation [28, 29]. Ryan and Deci [30] defined the former in their self-determination theory (SDT) as a type of motivation to attain distinct outcomes (i.e., to attain rewards or to avoid punishments). Two examples are an employee who works overnight to get overtime payments, and an engineer who works hard so that his or her family does not suffer. On the other hand, intrinsic motivation is defined as a type of motivation to do an activity because of the inherent enjoyment of the activity rather than to attain distinct outcomes. Moreover, adopted as part of the theory, DeCharms [31] pointed out that intrinsic motivation reflects a psychological need for competence and autonomy. The first refers to the sense of acquiring skills in activities that are optimally challenging, whereas autonomy refers to the sense that the behavior is authentic rather than internally intimidated or externally compelled. When these two needs are supported, intrinsic motivation may last, but when these needs are not satisfied, the intrinsic motivation is undermined. Furthermore, with regards to fatigue, Hockey [10], in his motivational control theory, states that self-initiated activities or tasks (i.e., with high intrinsic motivation) are unlikely to cause mental fatigue.

Intrinsic motivation, however, is often confounded with another type of motivation, namely, achievement motivation [32]. In their notion, they divide motivation into three types: intrinsic, achievement, and extrinsic. They argue that intrinsic motivation should be viewed as a type of motivation that arises from the enjoyment of doing an activity per se, and that it should be separated from other motives, e.g., to attain skills, to make progress, or to meet some quality standards. They emphasize that achievement motivation does not focus on the pleasure of the activity itself but of achieving new standards and keeping progress; for example, a doctor who wants to become even better at diagnosing patients. Therefore, to avoid confusion, a study of intrinsic motivation should solely focus on the enjoyment of doing the activity and attempt not to mix it with achievement motives such as competition [33].

Even though the effects of intrinsic motivation in prolonged tasks are evident in daily life, e.g., game players can play computer games for many hours regularly [34], we have not found any studies aimed at finding connections between mental fatigue and intrinsic motivation.

To investigate the links between intrinsic motivation and mental fatigue, we performed an experiment where we asked participants who liked Sudoku to solve Sudoku puzzles on a computer screen in two alternating conditions: low-level motivation (LL) and high-level motivation (HL). We hypothesized that if intrinsic motivation were an important component in mental fatigue, participants would be able to maintain performance and attention to the task in HL conditions over time. More specifically, individuals who like doing an activity because of the sense of satisfaction they receive from the activity itself would not show any effects of mental fatigue. On the other hand, in LL conditions, where the Sudoku involved less enjoyment, we predicted that performance would decline and be susceptible to distractions over time.

## 2 Method

### 2.1 Participants

Prior to the experiment, the sample size was calculated using G-power statistical software analysis [35]. The experiment was within-subject and designed to have a power of .90 (type II error = .10), a significance level of .05, and a large effect-size (d = .80) (based on a similar experiment from Herlambang et al. [9]). Therefore, the required sample size using these parameters was 19. To avoid problems with very small sample sizes, we decided to aim for a slightly larger sample of 30.

Thirty-two healthy university students joined the experiment and received monetary reward for participating. Four participants gave up during the experiment. Heart rate data were lost due to equipment problems in two participants. Therefore, the final sample consisted of 28 participants (17 male; mean age = 24.57 years, SD = 4.21), and 26 for heart rate variability (HRV) analysis. All participants included in the study gave written informed consent. The research was approved by the Research Ethics Committee of the University of Groningen (CETO-58444279), and it was in compliance with the 1964 Helsinki declaration.

### 2.2 Procedure

University students who liked playing Sudoku were invited to join our experiment. After registration, we asked how often they played Sudoku. If they indicated having played Sudoku at least five times within the last month, we asked them to solve a difficult Sudoku puzzle within six hours, which they received by email. If they did, they were eligible to participate.

A few days before the experiment, all participants received another email informing them of the details of the study. The email did not mention mental fatigue. It asked participants not to drink coffee 24 hours, not to consume heavy meals or perform any exercise an hour before the experiment started. Also, participants were required to have enough sleep. The email stated that participants were not allowed to participate if they had heart abnormalities.

On the day of the experiment, participants were seated 60 cm in front of an LCD monitor. They were asked to attach the heart rate monitor on their chest. In the case of chest hair, we asked them to shave it in order to attach the heart rate monitor properly. Afterward, we asked them to rest for five minutes and checked their resting heart rate: if their resting heart rate was normal (the heart rate does not go above 85 beats per minute, which is a threshold used to determine an indication of cardiovascular disease) [36], they were allowed to proceed to the next step.

Next, they were asked to hand over their wristwatches, turn off their phones, and sign an informed consent form. Afterward, they were requested to put their chin on a chin rest of the eye-tracker, and we performed calibration and drift correction before the experiment started. During the experiment, participants were not allowed to move, except when they felt tired, but they had to remain seated in the chair.

Before the main experiment, participants performed a practice session to familiarize them with the Sudoku until they were ready to proceed (max. 15 min). On the left side of the table, we put 14 sheets of subjective ratings to be filled in every time a block ended and asked them to put it on their right side when finished with a block.

After the experiment finished or when participants decided to give up, they had a debriefing session, in which we explained the purpose of the study.

### 2.3 Task

**Fig 1:**
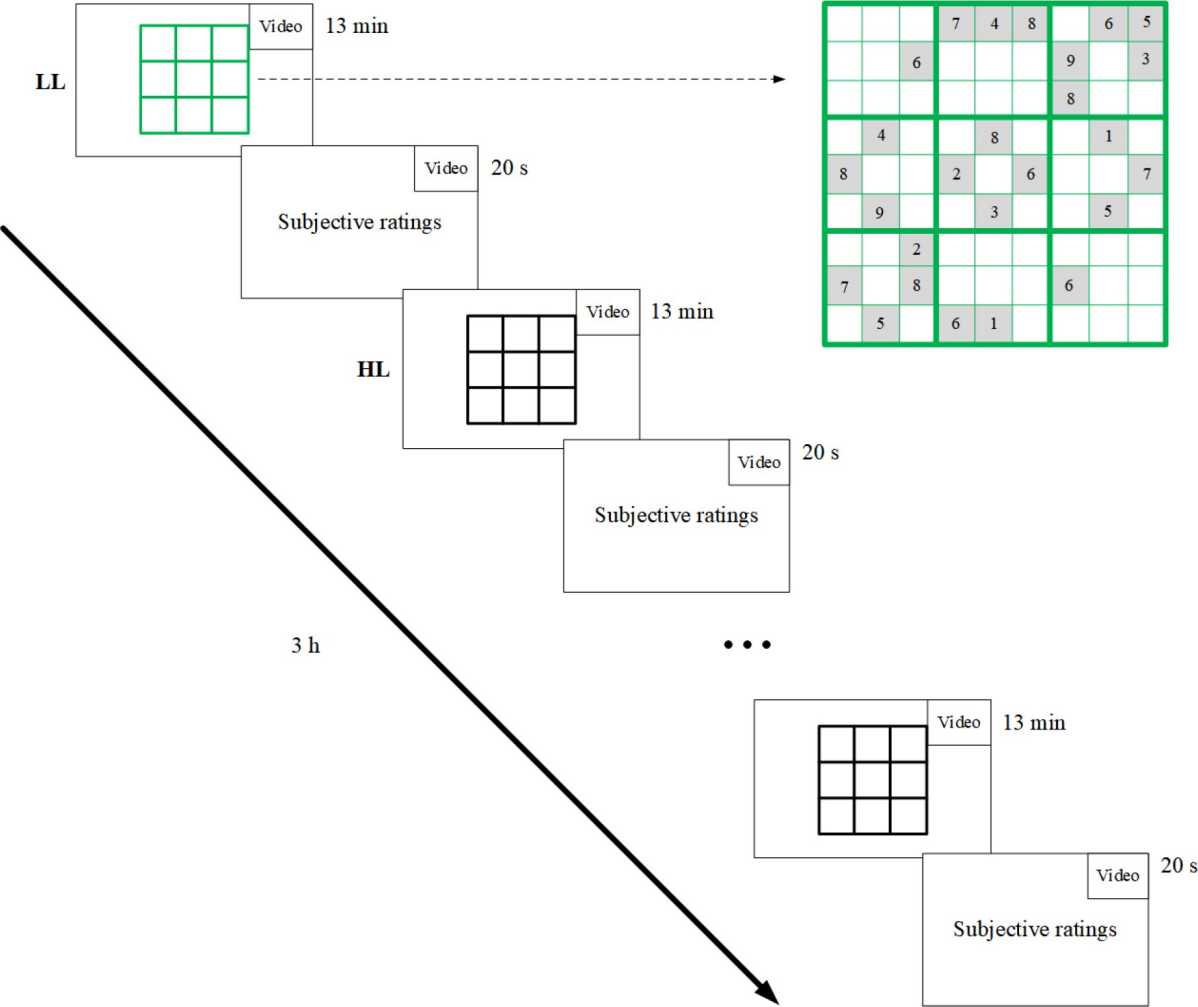
The flow of the experiment. The LL Sudoku (LL) is indicated by green edges. Initial filled-in cells had a gray-colored background. After a block ends, it continues to a screen which asks participants to fill in the subjective ratings. A video distractor is played continuously in the top right of the screen.

Participants were asked to play Sudoku puzzles on a computer screen continuously for three hours without rests. The experiment consisted of 14 blocks of two alternating conditions: low-level (LL) intrinsic motivation in the odd blocks and high-level (HL) intrinsic motivation in the even blocks. The duration for each block was 13 min. To indicate the two different conditions, the Sudoku puzzles in the LL blocks were drawn in green, whereas in the HL blocks, they were drawn in black. After a block ended, the computer screen proceeded to a subjective-ratings screen (Fig 1).

The Sudoku puzzles had 3 x 3 boxes, and each box consisted of 3 x 3 cells; therefore, the total number of cells was 81. At the start of the puzzle, a number of cells already had numbers in them between one and nine. The task of the participants was to fill in the remaining cells with numbers. Each box should include all the numbers from one to nine, and each number should only occur once in a row and once in a column.

To solve the Sudoku, participants were provided with a mouse. Participants had to click on an empty cells in the Sudoku and were then shown several buttons of numbers from one to nine (from left to right) positioned in the center of the screen, and then pressed one of the numbers. If the answer was incorrect, participants would hear a ‘beep’ sound, and the cell would remain empty. If the answer was correct, there was no sound, and the cell would be filled in with the chosen number.

In the LL condition (the odd blocks), a new Sudoku puzzle was generated every five trials, that is, after five clicks within the Sudoku box. After 13 min had elapsed, participants had to fill in three subjective rating scales on a sheet of paper for 20 s. Participants then continued with a new block. In HL blocks, if participants could not complete a Sudoku puzzle after 13 min, they continued solving the same Sudoku puzzle in the next HL block. In contrast, if participants were able to finish a Sudoku puzzle in a block, the algorithm would generate a new Sudoku puzzle immediately. This design assumed that being able to finish a complete puzzle was more motivating than just solving a few steps. It allowed for more long-term planning and, therefore, a more motivating mental investment in the task.

### 2.4 Materials

We used the Eyelink Duo from SR Research to obtain pupil diameter, eyeblinks, gaze positions, and saccades with a sample rate of 250 Hz by measuring participants’ dominant eye during the experiment. To measure heart rate, we used the Cortrium C3 holter monitor. The device has been tested and is valid and realiable to be used as an instrument to measure heart rate [37, 38].

For subjective measures, we printed the Rating Scale Mental Effort (RSME), the National Aeronautics and Space Administration Task Load Index (NASA-TLX), and the Visual Analog Scale (VAS) on a two-sided page with RSME as the first measure on the first page followed by NASA-TLX and VAS as the second and third measure on the second page.

The display used in the experiment was a 19-inch square LCD monitor. We played Simon’s cat video, a black-and-white animation of a cat, continuously with a resolution of 320 x 180 in the top right of the screen as a distractor. We were granted to use the video by Simon’s Cat Ltd.

To present the Sudoku puzzles to participants, we used OpenSesame with a resolution of 1,280 x 960 [39] and PyGaze [40] to give commands to the eye-tracker. The puzzles were designed to be equally difficult in both conditions. To generate a new puzzle, we used constraint propagation and searching algorithms [41] and filled in 26 cells randomly (see Fig 1). All puzzles were solvable, regardless of condition. The difficulty to solve the puzzles was chosen as moderate.

### 2.5 Measures

#### 2.5.1 Subjective measures

We used VAS to measure the subjective feeling of fatigue. This instrument has high validity and reliability to measure fatigue [14]. In addition, we used RSME to measure mental effort [42], and NASA-TLX to measure physical load, mental load, temporal load, frustration level, performance and effort [43, 44]. Both RSME and NASA-TLX are reliable and valid to be used as subjective assessments of effort and mental workload respectively [45, 46].

For the first seven participants, we used NASA-TLX as a measure of workload and effort. Later, we added RSME due to lack of sensitivity of the effort scale of the NASA-TLX. Therefore, RSME data were complete for only 21 participants.

#### 2.5.2 Performance measures

We measured reaction time (RT) as the time between mouse clicks within the Sudoku puzzle. A click outside the Sudoku was not considered a response, and RT was not recorded. We excluded the response time of the first click of each Sudoku puzzle from the analysis because of its high variability. In addition, we measured the number of clicks for each block. To measure accuracy, we expressed it as the percentage of correct clicks for each block.

#### 2.5.3 Physiological measures

Heart rate variability (HRV) is the variability between consecutive heartbeats and reflects how individuals react to environmental and internal changes [47, 48]. HRV provides information on individuals' autonomous nervous system over time [49, 50]. Moreover, the mid-frequency (MF) band of HRV (0.07–0.14 Hz) is commonly used as a measure of mental effort [51, 52]. HRV is a valid and reliable measure to measure mental effort [53]. It is a non-invasive method to continuously monitor individuals’ physiological condition. In this experiment, participants were predicted to exert mental effort differently in LL and HL conditions, and an increase in effort is suggested to correlate with high motivation [26]. Therefore, measuring participants' mental effort using the MF band of HRV could provide information on how participants performed and responded to the experimental manipulation over time.

Raw data of the heart rate signal from the Cortrium C3 were preprocessed using PreCAR to detect and correct R-peak artefacts. Afterward, we used CARSPAN [54] to determine heart rate variability in the MF band. Power data for each block were normalized by dividing the power of each block by the average power across the experiment.

Pupillometry used in this study consisted of several measures: pupil diameter, eyeblinks, gaze positions, and saccades. We obtained raw data of the pupillometry from Eyelink Duo. Next, we used EDF2ASC (a software package from SR Research) to convert the raw data to ASCII format and used Eyelinker [55] to convert ASCII format to a more structured format to be analyzed in R (Version 3.4.2). We filtered all pupillometry data from the start of each block to the end of the block.

Pupil diameter is commonly used to measure cognitive load and control [56]. In addition, a recent study of mental fatigue used pupil dilation to measure task engagement in which the pupil dilates when participants re-engaged with a task motivated by extrinsic rewards [11]. In this experiment, we measured the exploitation-exploration effect (the engagement-disengagement effect) on pupil dilation in LL and HL conditions. We normalized pupil diameter for each block by dividing the average of that block by the average of the entire experiment.

Eyeblinks have been used as a measure of fatigue and workload [57], and we used eyeblinks to measure mental fatigue in this experiment. We predicted that eyeblinks would increase over time regardless of conditions in the experiment. For data analysis, we calculated the mean of eyeblink frequency and eyeblink duration.

To measure distractibility by a video distractor, i.e., Simon’s cat video, we used eye gaze positions. We hypothesized that a decrease in motivation leads to more distractions [11, 58]. We predicted that if the intrinsic motivation were essential to keep participants engaged (i.e., stay motivated) with the Sudoku puzzles, they would be less distracted in HL conditions than in LL conditions. Each time the coordinate of eye gaze was within the cat video for at least 200 ms, we used it as an instance of visual distraction. This was based on our previous study [9], and the duration of 200 ms is also regarded as the average duration of eye fixations during reading [59]. For each block, we calculated the mean of visual distraction frequency and visual distraction duration.

To measure attention to the task, we used in-task eye saccades, i.e., when the starting and ending point of saccades were within the Sudoku puzzle. We assumed that participants made saccades movement frequently to solve Sudoku puzzles, searching for the right number for the right cell. Afterward, we calculated the mean of saccades frequency and saccades amplitude.

#### 2.5.4 Statistical analysis

We used linear mixed-effects models for all measures by using Lme4 package [60] of R (Version 3.4.2). For visual distraction frequency analysis, we applied log-transformation because the dataset was not normally distributed. Moreover, we used the Car package in R to obtain *p* values [61].

To determine the best fitting model, we compared Akaike criteria from the simplest model to more complex models and used the function *anova* in R. First, we compared time-on-task with condition; both are fixed effects in the models. All models used participants as the random effect. Next, we compared the chosen model with a model of interaction between time-on-task and condition. In addition, we examined the residuals and fitted values to comply with the assumption of constant variance.

## 3 Results

Tables show the best-fitted model in each measure, regardless of significance.

### 3.1 Subjective measures

#### 3.1.1 Fatigue

**Fig 2:**
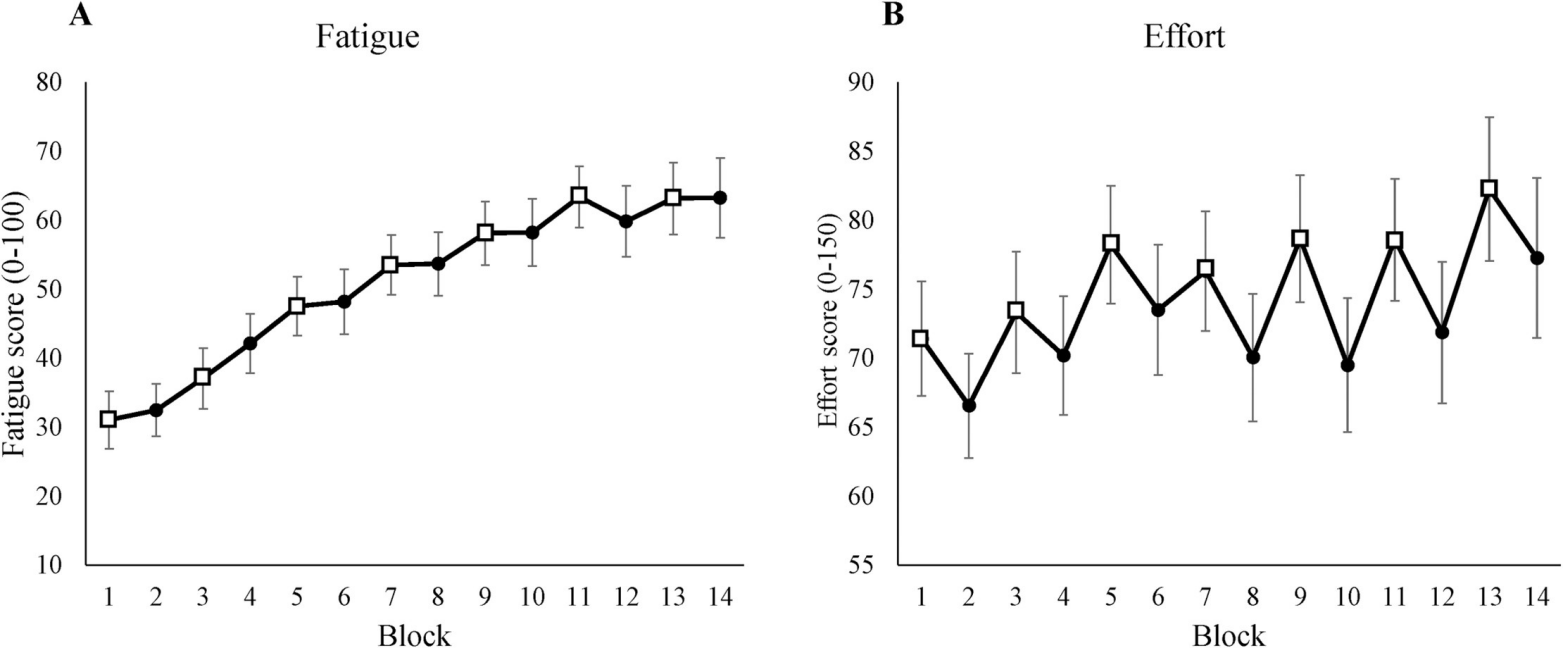
The subjective measure of fatigue and effort. (A) Average fatigue scores for each block using the VAS. The y-axis shows subjective fatigue scores from 0 to 100. (B) Average effort ratings for each block using the RSME. The y-axis shows the subjective mental effort scores from 0 to 150. All x-axes show blocks, where odd blocks represented by square markers are the low motivation blocks. Error bars in each block represent standard errors.

To check our fatigue manipulation, we used the Visual Analog Scale (VAS). Time-on-task had a significant effect on the fatigue score (Table 1), which increased linearly from the first to the last block (see Fig 2A). Including condition as a fixed effect did not improve the model.

**Table 1:** The mixed-effect result of the subjective measure of fatigue and effort from the best fitted model.

Measure					95% Confidence interval	
	Variable	Mean	Standard error	*p* Value	Lower limit	Upper limit
Fatigue	Intercept	3.11	0.41			
	Time	0.26	0.01	<0.001**	0.23	0.29
Effort	Intercept	72.27	4.55			
	Time	0.67	0.21	0.001*	0.26	1.07
	Condition	-6.37	1.66	<0.001**	-9.63	-3.11

* *p*<0.05

**p<0.001.

#### 3.1.2 Effort

To measure subjective mental effort, we used the Rating Scale Mental Effort (RSME). Table 1 shows that both time-on-task and condition had a significant effect on subjective mental effort. Fig 2B shows that participants reported higher ratings in low-level motivation (LL) blocks, and that subjective mental effort increased over time. Including the interaction as a fixed effect did not improve the model. In addition, the RSME score showed a significant correlation with the frustration scale of NASA-TLX *r*(12) = .86, *p* < .01.

#### 3.1.3 NASA-TLX

**Fig 3:**
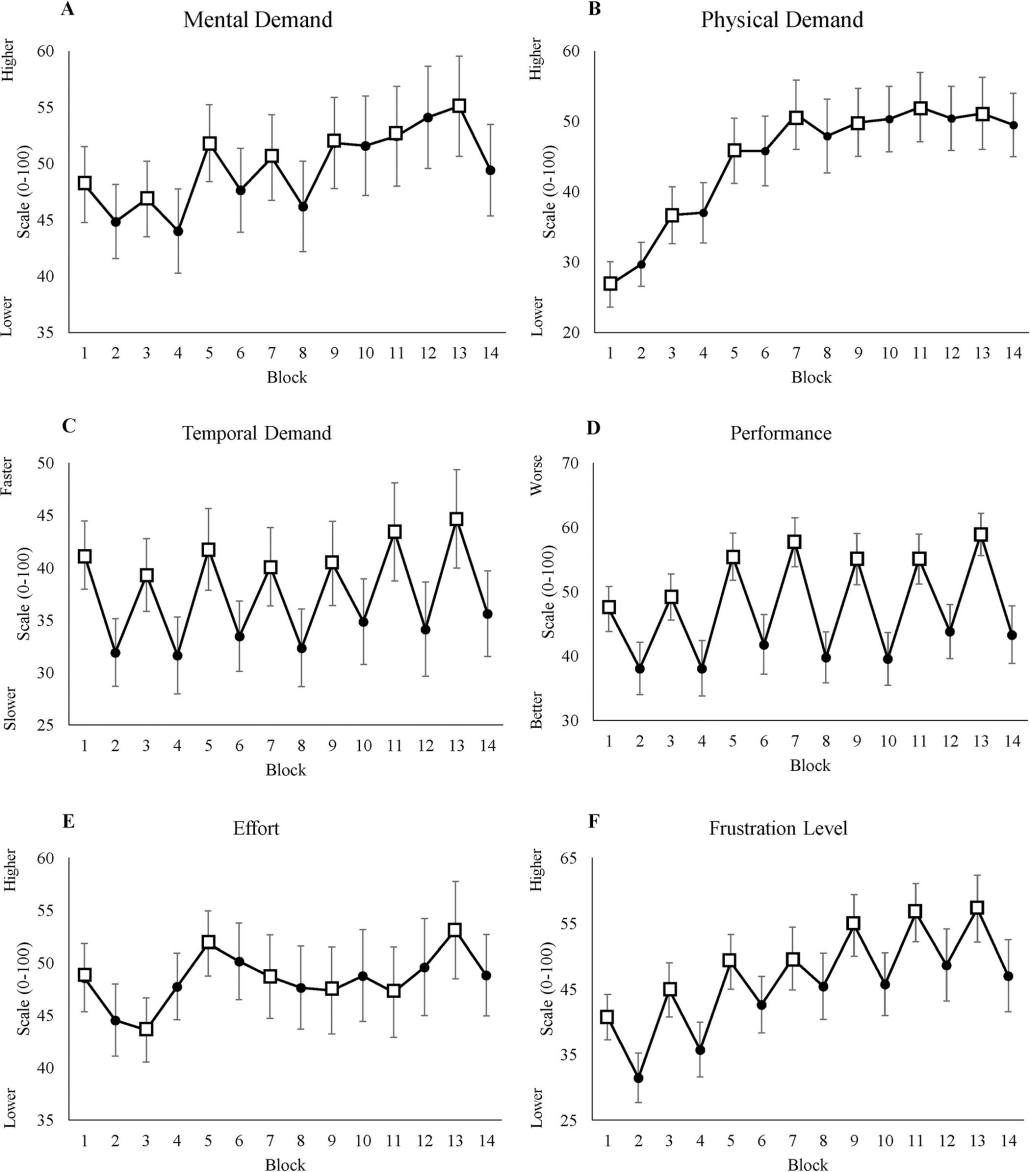
NASA-TLX scales. (A) Average score of mental demand for each block. (B) Average score of physical demand for each block. (C) Average score of temporal demand for each block. (D) Average score of performance for each block. (E) Average score of subjective effort for each block. (F) Average score of frustration level for each block. All figures’ y-axes show the score for each scale from 0 to 100, and x-axes show blocks, where odd blocks represented by square markers are the low motivation blocks. Error bars in each block represent standard errors.

Table 2 shows that time-on-task had a significant effect on mental demand, physical demand, performance, and frustration level. In addition, Fig 3A, 3B, 3D and 3F show that all these dimensions increase over time. Moreover, the effect of condition on mental demand, temporal demand, performance, and frustration level was also significant. Participants reported higher frustration levels in LL blocks. However, we did not find any significant effect on the effort scale, which is not shown in the table.

**Table 2:** The mixed-effect results of NASA-TLX from the best fitted model.

					95% Confidence interval	
Scale	Variable	Mean	Standard error	*p* Value	Lower limit	Upper limit
Mental Demand	Intercept	46.59	3.53			
	Time	0.65	0.13	<0.001**	-5.41	-1.23
	Condition	-3.32	1.06	0.001*	0.36	0.88
Physical Demand	Intercept	31.53	4.15			
	Time	1.74	0.14	<0.001**	1.45	2.02
Temporal Demand	Intercept	41.55	3.15			
	Condition	-8.12	1.28	<0.001**	-10.64	-5.59
Performance	Intercept	49.65	3.01			
	Time	0.63	0.21	0.002*	0.22	1.04
	Condition	-14.09	1.67	< .001**	-17.38	-10.81
Frustration Level	Intercept	40.78	3.95			
	Time	1.37	0.18	<0.001**	1.01	1.73
	Condition	-9.41	1.48	<0.001**	-12.33	-6.48

* *p*<0.05

**p<0.001.

### 3.2 Performance measures

#### 3.2.1 Response time

**Fig 4:**
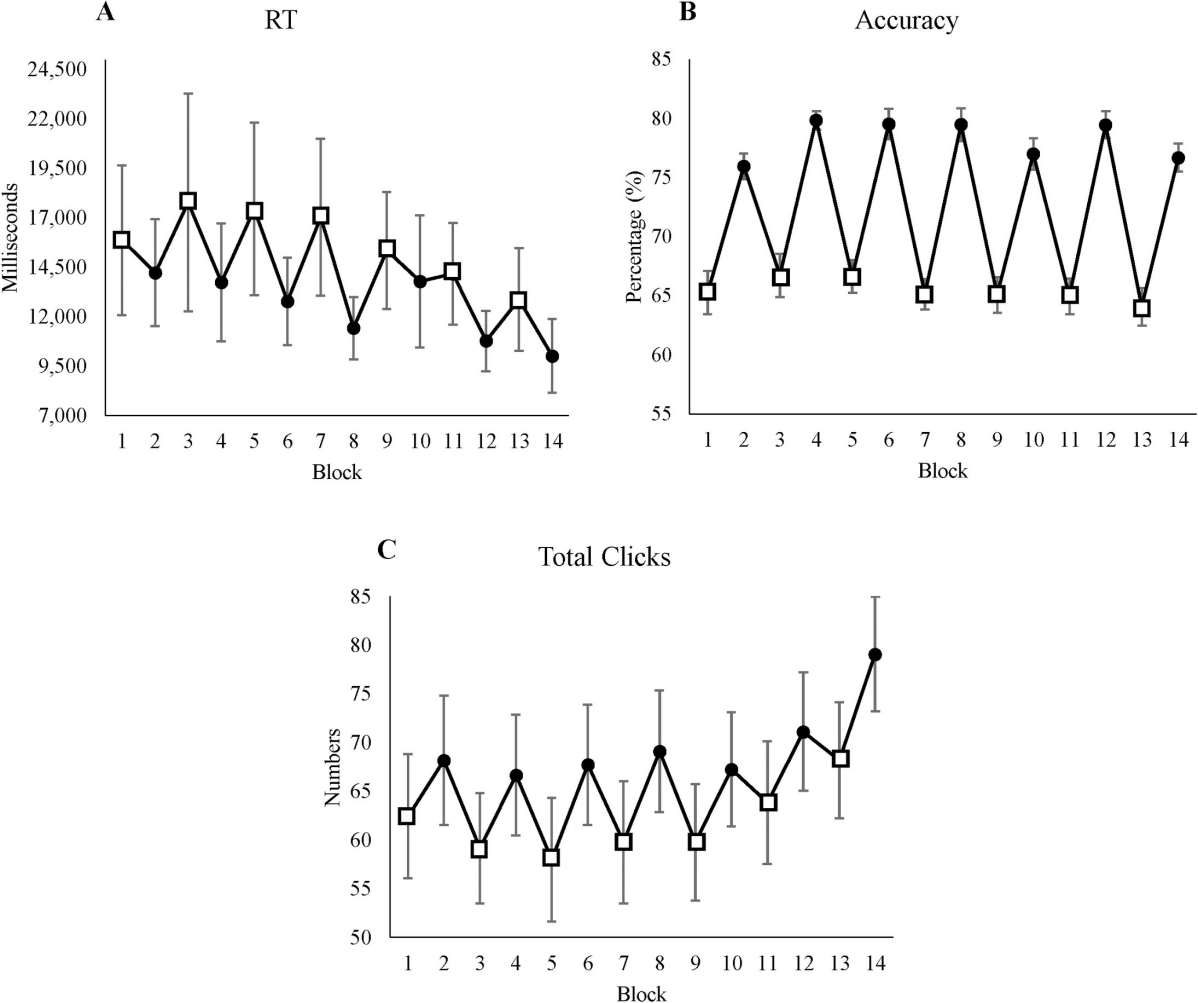
Performance measures. (A) Average response time for each block. (B) Average accuracy for each block. (C) The number of clicks for each block. All figures’ y-axes show their value respectively, and x-axes show blocks, where odd blocks represented by square markers are the low motivation blocks. Error bars in each block represent standard errors.

Response time decreased significantly over time (Table 3). In addition, RTs were significantly slower in LL blocks and faster in HL blocks (see Fig 4A). Including the interaction between time-on-task and condition did not improve the model.

**Table 3:** The mixed-effect result of performance measures from the best fitted model.

					95% Confidence interval	
Measure	Variable	Mean	Standard error	*p* Value	Lower limit	Upper limit
RT	Intercept	18,031. 01	2,765.88			
	Time	-320.91	123.86	0.009*	-564.31	-77.49
	Condition	-3,078.78	998.61	0.002*	-5,041.19	-1,116.38
Accuracy	Intercept	65.41	0.87			
	Condition	12.88	0.62	<0.001**	11.64	14.11
Total clicks	Intercept	57.23	5.78			
	Time	0.62	0.14	<0.001**	0.32	0.91
	Condition	7.67	1.21	<0.001**	5.31	10.03

* *p*<0.05

**p<0.001.

#### 3.2.2 Accuracy

We found a significant effect of condition on accuracy. Including time-on-task as a fixed effect did not improve the model (Table 3). Accuracy was lower in LL blocks and higher in HL blocks (see Fig 4B).

#### 3.2.3 Total clicks

This measure shows the number of clicks for each block regardless of accuracy. Time-on-task had a significant effect on total clicks, where participants made more clicks over time (Table 3). In addition, the effect of condition was significant, where participants made more clicks in the HL condition (see Fig 4C). Including the interaction did not improve the model.

### 3.3 Physiological measures

#### 3.3.1 HRV—MF band

**Fig 5:**
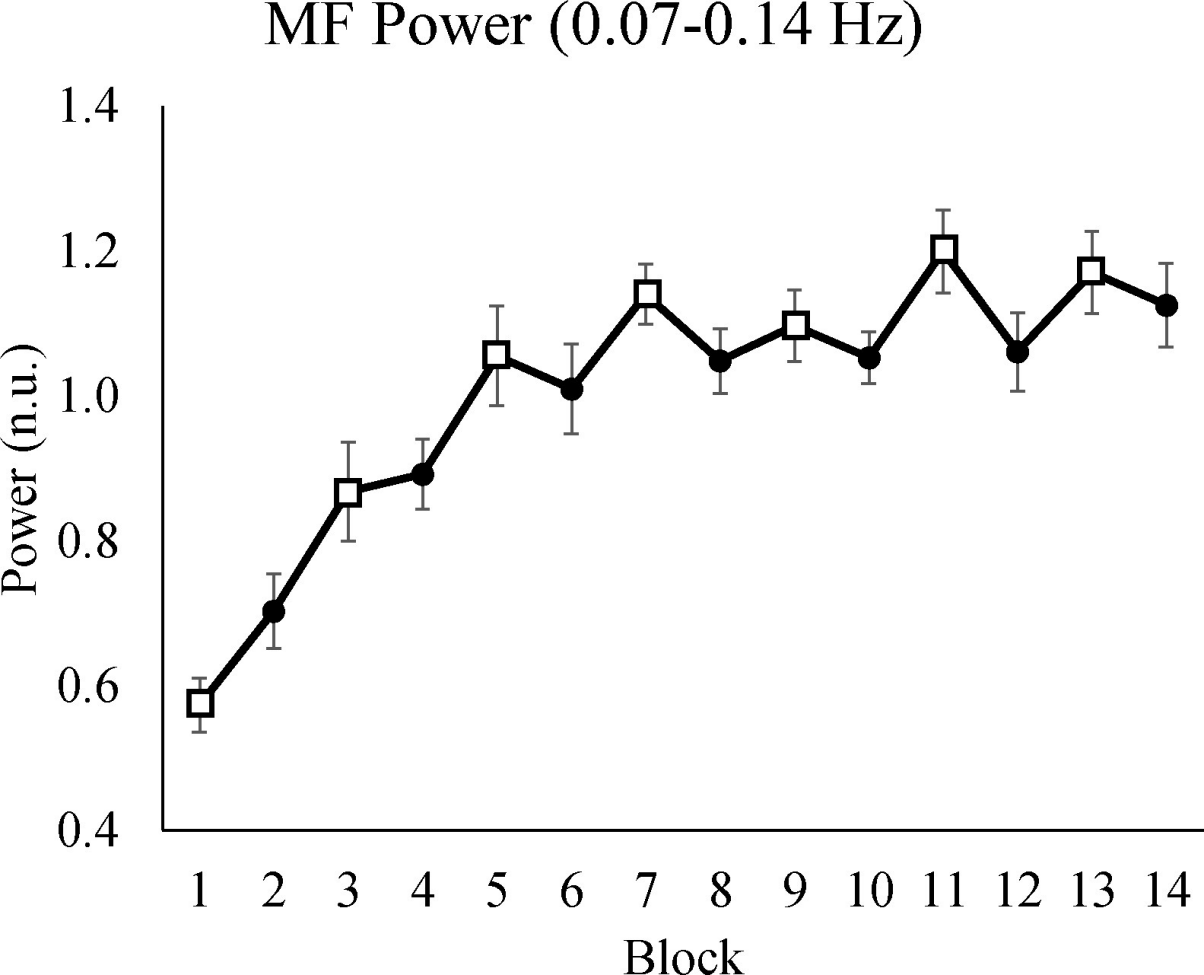
Average power of HRV in the MF band. The y-axis shows the normalized value of the MF power. The x-axis shows blocks, where odd blocks represented by square markers are the low motivation blocks. Error bars in each block represent standard errors.

To measure cognitive mental effort, we calculated the power in the MF band of HRV and expressed power in a block as a percentage of the average power of the whole experiment. Higher power in the MF band indicates that participants invested less mental effort [51, 52]. Conversely, lower power indicates the opposite. Table 4 shows that time-on-task had a significant effect on MF power, which indicates that participants invested less mental effort over time. In addition, the interaction between time-on-task and condition was significant: The difference between the two conditions increased over time (see Fig 5).

**Table 4:** The mixed-effect result of MF power from the best fitted model.

				95% Confidence interval	
Variable	Mean	Standard error	*p* Value	Lower limit	Upper limit
Intercept	0.71	0.04			
Time	0.04	0.01	<0.001**	0.03	0.05
Condition	0.04	0.06	0.459	-0.07	0.17
Time x Condition	-0.01	0.01	0.039*	-0.03	-0.01

* *p*<0.05

**p<0.001.

#### 3.3.2 Pupil diameter

**Fig 6:**
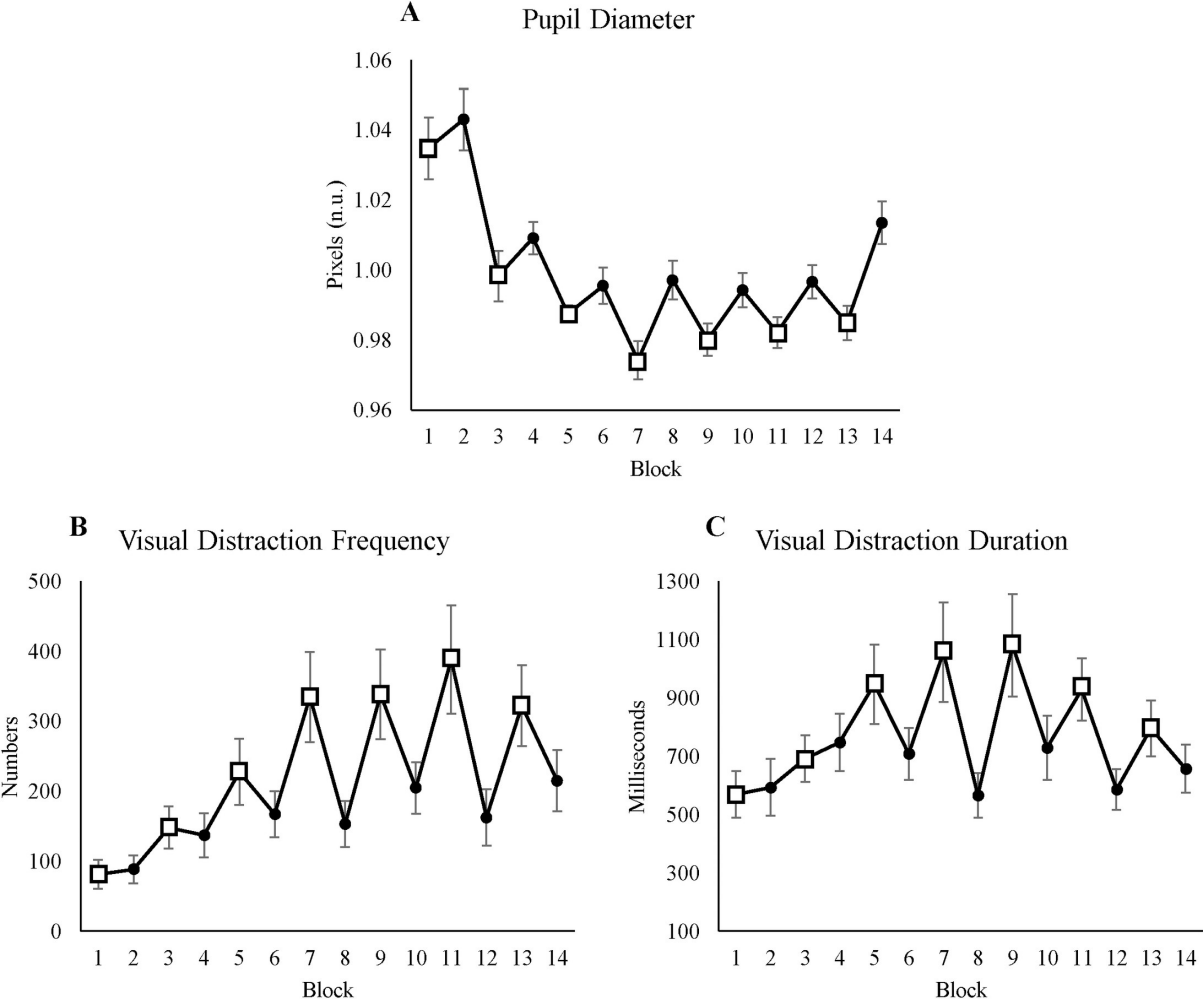
Pupil diameter and visual distraction. (A) Average pupil diameter for each block. (B) Average visual distraction frequency for each block. (C) Average visual distraction duration for each block. All figures’ y-axes show their value respectively, and x-axes show blocks, where odd blocks represented by square markers are the low motivation blocks. Error bars in each block represent standard errors.

To measure the involvement of cognitive control, we used pupil diameter. Time-on-task showed a significant effect on pupil diameter (see Table 5). Fig 6A shows that pupil diameter decreased over time, indicating lower cognitive control over time. Furthermore, the effect of condition on pupil diameter was significant, with the pupil diameter dilating more in HL blocks, indicating higher cognitive control. Including the interaction between time-on-task and condition did not improve the model.

**Table 5:** The mixed-effect result of pupillometry measures from the best fitted model.

					95% Confidence interval	
Measure	Variables	Mean	Standard error	*p* Value	Lower limit	Upper limit
Pupil diameter	Intercept	1.01	0.01			
	Time	-0.01	0.01	<0.001**	-0.02	-0.01
	Condition	0.02	0.01	<0.001**	0.01	0.03
Visual distraction frequency	Intercept	1.70	0.11			
	Time	0.05	0.01	<0.001**	0.04	0.07
	Condition	-0.01	0.09	0.946	-0.18	0.16
	Time x Condition	-0.03	0.01	0.002*	-0.05	-0.01
Visual distraction duration	Intercept	706.16	92.02			
	Time	22.98	8.79	0.008*	5.71	40.25
	Condition	-35.47	105.87	0.737	-243.52	172.57
	Time x Condition	-24.93	12.43	0.044*	-49.35	-0.49
Eyeblink frequency	Intercept	181.78	27.92			
	Time	6.05	0.61	<0.001**	4.86	7.25
Eyeblink duration	Intercept	232.77	70.76			
	Time	4.69	2.37	0.047*	0.03	9.35
Saccades frequency	Intercept	1,876.42	54.64			
	Time	-23.23	4.16	<0.001**	-31.41	-15.04
	Condition	-65.65	50.15	0.191	-164.22	32.91
	Time x Condition	19.18	5.89	0.001*	7.61	30.76
Saccades amplitude	Intercept	3.19	0.07			
	Condition	-0.14	0.02	<0.001**	-0.18	-0.11

* *p*<0.05

**p<0.001.

#### 3.3.3 Visual distraction frequency

We used visual distraction frequency to measure how often participants shifted their attention to the video distractor. Time-on-task had a significant effect on visual distraction frequency (see Table 5), where participants watched the cat video more often over time. In addition, time and condition had a significant interaction effect on visual distraction frequency: Over time, visual distraction frequency increased more in LL blocks than in HL blocks (see Fig 6B).

#### 3.3.4 Visual distraction duration

Visual distraction duration measured how long participants watched the cat video. Time-on-task had a significant effect on visual distraction duration (see Table 5), where over time participants watched the cat video longer. Moreover, time-on-task and condition had a significant interaction effect on visual distraction duration. Fig 6C shows the interaction between time-on-task and condition. Visual distraction duration remained relatively stable in HL blocks, but it increased over time in LL blocks.

#### 3.3.5 Eyeblink frequency

**Fig 7:**
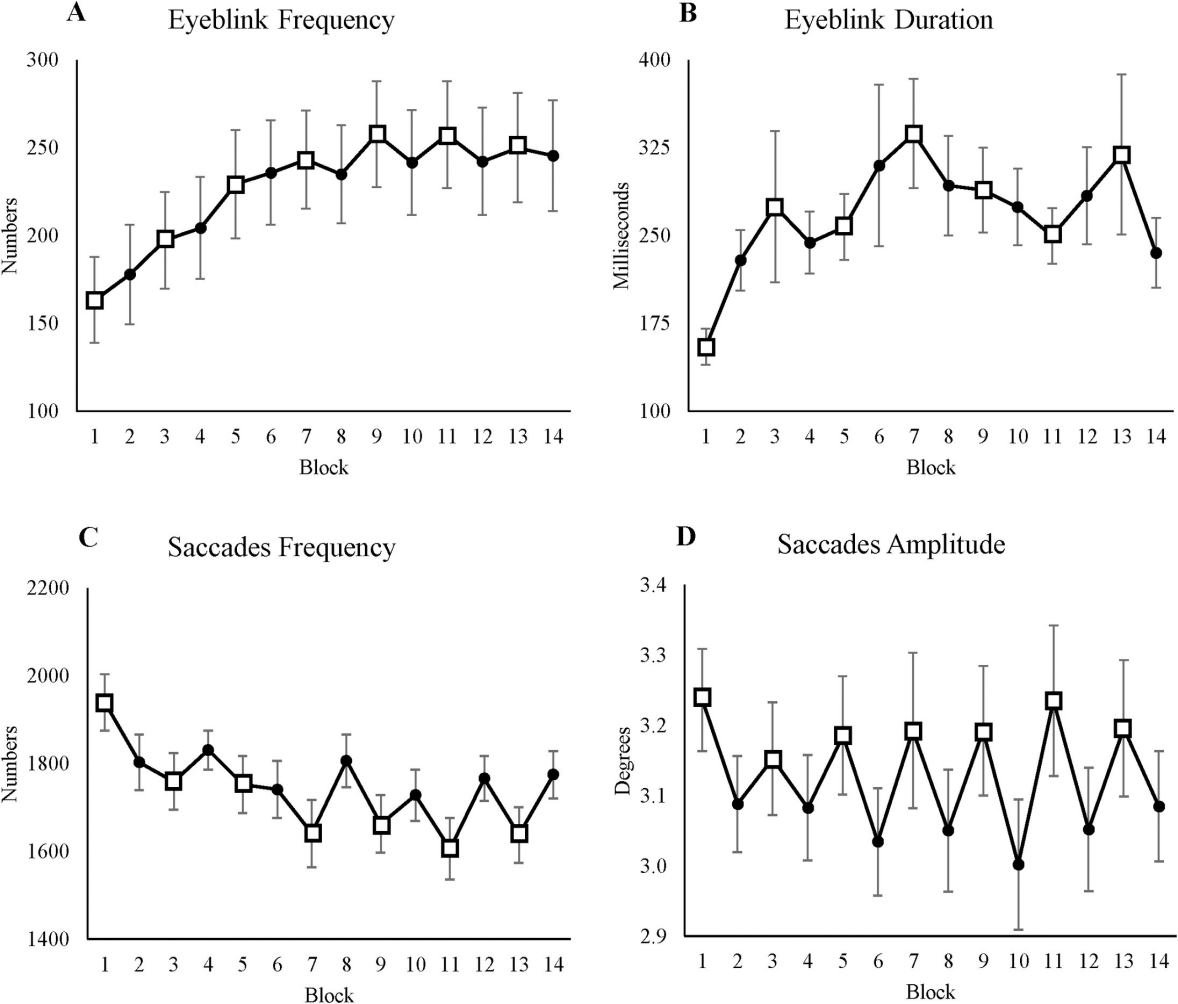
Eyeblinks and saccades. (A) Average eyeblink frequency for each block. (B) Average eyeblink duration for each block. (C) Average saccades frequency for each block. (D) Average saccades amplitude for each block. All figures’ y-axes show their value respectively, and x-axes show blocks, where odd blocks represented by square markers are the low motivation blocks. Error bars in each block represent standard errors.

We used eyeblink frequency as an indicator of fatigue. Table 5 shows that time-on-task had a significant effect on eyeblink frequency, with participants blinking more often over time (see Fig 7A). Including condition as a fixed effect did not improve the model.

#### 3.3.6 Eyeblink duration

The purpose of eyeblink duration was similar to that of eyeblink frequency. Eyeblink duration significantly increased (see Table 5 and Fig 7B); including condition as a fixed effect did not improve the model.

#### 3.3.7 Saccades frequency

We used saccades frequency to measure participants’ attention to solving Sudoku puzzles. Saccades frequency significantly decreased over time (see Fig 7C**)**. In addition, time-on-task and condition had a significant interaction effect on saccades frequency: Over time, the difference between the two conditions increased (see Table 5).

#### 3.3.8 Saccades amplitude

To measure participants’ attention towards the task, we used saccades amplitude. Participants made significantly smaller saccade movements in HL blocks (see Table 5 and Fig 7D), suggesting that participants were more careful in HL blocks, searching for the right number for the right cell. Including time-on-task did not improve the model.

## 4 Discussion

### 4.1 Hypothesis & results

In this study, we investigated the effect of intrinsic motivation on mental fatigue. We hypothesized that participants who liked playing Sudoku would not show effects of mental fatigue doing the task, particularly in high-level motivation (HL) blocks.

Several measures showed the effects of our manipulation of intrinsic motivation on performance and supported our hypothesis that intrinsic motivation helped participants maintain performance. The subjective measure of fatigue (i.e., VAS) showed that participants reported becoming fatigued over time regardless of condition, which suggested that our fatigue manipulation was successful. However, even though participants felt fatigued, they were able to maintain performance and attention in HL blocks.

Accuracy remained at the same level in HL blocks but was lower in LL blocks. The difference in the accuracy between the two conditions may lie in the fact that participants were more inclined to use a guessing strategy in the LL blocks. In contrast to paper-and-pencil Sudoku, where guessing is a suboptimal strategy, in our experiment, guessing could be beneficial to participants, as they received immediate feedback after choosing a number: Participants could still solve the puzzle with no increase in effort. Although accuracy was lower in LL blocks, it did not decrease over time (it remained at this low level). Therefore, the increase in subjectively reported mental fatigue (VAS) did not lead to decreased performance over time.

Moreover, in HL blocks, participants were less distracted (visual distraction frequency and visual distraction duration were lower), more attentive toward the task (saccades frequency was more frequent) and more conscientious to solve the puzzles (saccades amplitude was smaller). In contrast, participants gradually became more susceptible to distractions and less attentive to the task in LL blocks. Also, the MF power of HRV suggests that participants invested more mental effort in HL blocks, whereas in LL blocks, they invested less mental effort. This result is in line with the motivation intensity theory [27]. Because success was not possible in the LL blocks, the exerted effort in these blocks would be lower compared with HL blocks. On the other hand, the exerted effort was high in HL blocks, since success was possible and beneficial in these blocks (see Wright et al. [26]).

One may argue that the duration of the task affected the way participants regulated their effort: Since they knew that the task would take a few hours to complete, they started to work slower in the first few blocks and became faster in the last few blocks, as compared to if the task length would be shorter in time. However, it depends on how they evaluated the costs and benefits of performing the task over time. Therefore, participants will not perform best (not exerting the necessary effort to perform a task) if they perceive a short-duration task to be non-rewarding. On the other hand, they will keep exerting effort if they perceive a task to be rewarding, regardless of duration (see Hockey [58]). In this study, participants exerted more mental effort in HL blocks because they perceived the task in these blocks to be more enjoyable, rewarding, and feasible.

Furthermore, regarding effort, RSME, the MF power of HRV, and the effort scale of NASA-TLX showed different effects. Participants reported higher RSME effort in LL blocks, but the power in the MF band physiologically suggests that participants exerted less mental effort in these conditions. It is not uncommon to find disagreement between subjective and physiological measures [62]. A possible explanation for this difference may lie in participants’ difficulty in rating subjective effort as a separate entity. In LL blocks, the Sudoku puzzles were less satisfying than in HL blocks because participants had to reorient themselves with a new puzzle every five trials in these blocks. Since they knew that the puzzles would be tedious, and they needed to stay alert until the experiment ended, they may have rated this as increased effort on the RSME scale. Moreover, Veltman and Gaillard [63] already reported that RSME was more sensitive to measure mental effort than NASA-TLX, which may explain why the effort scale of NASA-TLX and RSME showed different effects in this experiment.

An interesting measure in this experiment was pupil diameter, which was larger in the first blocks, indicating a higher level of cognitive control in the first blocks. It has been suggested that a large pupil diameter indicates exploitation of the task, i.e., finding out how the task works [11]. We believe that although participants had experience with solving Sudoku puzzles, they still needed some time to fully understand how to do the task on the computer. In contrast to normal paper-and-pencil Sudoku, where people can write several candidate numbers in a cell, this was not possible here on the computer screen. This required finding a slightly different strategy, which was reflected in the larger pupil diameters in the first few blocks.

In addition, RTs decreased over time in both conditions. This can probably be attributed to a learning effect, which obscures any possible effects of fatigue on performance. As a result, participants had more chances to solve the Sudoku within a block, which was reflected in an increase in the total clicks over time.

It is possible to explain the results of this study using the resource theory. As part of the theory, rest can help individuals to recharge resources and thus maintain performance [4, 5, 64]. Since the accuracy in LL blocks remained stable, participants might have used these blocks to rest and restore their resources. Another possibility was that participants used the moment when they filled in subjective measures (i.e., 20 s after each block ended) to recover. However, the explanation of the resource account assumes that the recovery takes place within a short amount of time and can occur any time regardless of task duration.

With regard to the task design, we wanted to avoid that intrinsic motivation was confounded with achievement motivation (a sense of progress and motives to achieve particular standards [32]). Therefore, we avoided instructions that would make participants perceive the game as a competition, or make them believe they needed to meet a particular standard: Participants performed the experiment freely, could give responses anytime without restriction, and were able to stop trying at any moment during the experiment. We reasoned that low-level motivation (LL) blocks lowered intrinsic motivation, even in people who liked playing Sudoku, since only being able to perform a few steps makes it impossible to do long-term planning. On the other hand, in high-level motivation (HL) blocks, the task was designed to be engaging and enjoyable, and participants should enjoy doing the task. This was confirmed by the lower frustration ratings on the NASA-TLX in HL blocks.

### 4.2 Extrinsic and intrinsic motivation

Many studies have tried to find links between mental fatigue and motivation [24]. However, these studies were limited to a single type of motivation, namely extrinsic motivation. To investigate whether intrinsic motivation has the same effects as extrinsic motivation, we compared the results of this study with the results of our previous study in which we incorporated extrinsic rewards in the experiment [9].

In our previous study, we asked participants to perform a working memory task for 2.5 hr in two alternating conditions: reward and nonreward. In the reward condition, participants were offered monetary rewards for good performance. On the other hand, participants performed the experiment normally in the nonreward condition. In the previous study, we also played Simon’s cat video continuously as a distractor in the top right of the screen.

Both our studies had similar setups: Both experiments consisted of 14 blocks in which odd blocks were low motivation blocks (nonreward blocks in the previous study and LL blocks in the present study), and even blocks were high motivation blocks. In both experiments, we used three different measures: subjective, performance, and physiological measures. We compared several measures which were identical in both experiments: a subjective measure of fatigue, the MF power of HRV, pupil diameter, and visual distraction frequency.

**Fig 8:**
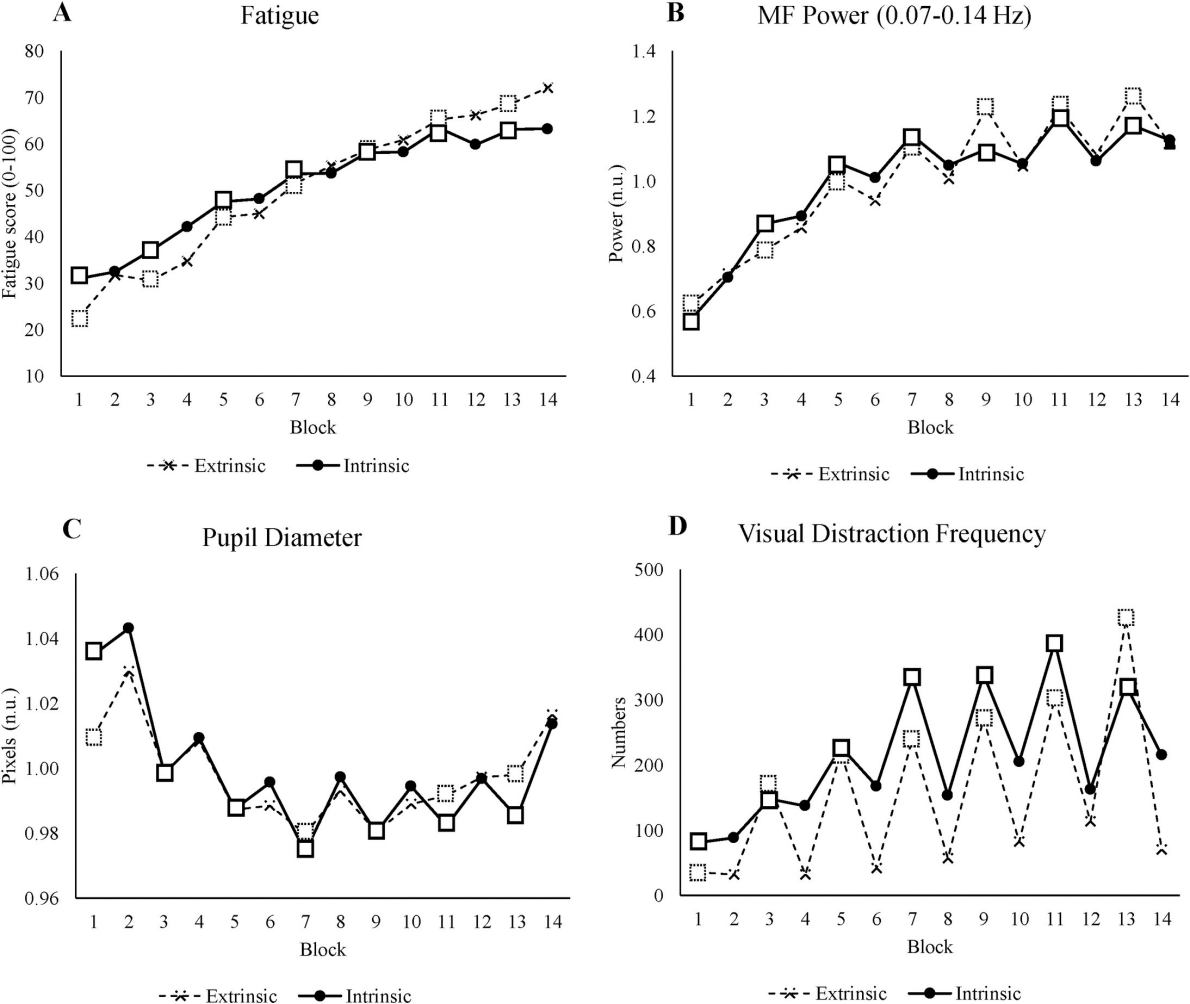
The results comparison between the study of the extrinsic motivation experiment represented by a dashed line and the intrinsic motivation experiment represented by a solid line. (A) Fatigue scale (B) MF power of HRV (C) Pupil diameter (D) Visual distraction frequency. All x-axes show blocks, where odd blocks represented by square markers are the low motivation blocks (nonreward condition in the extrinsic motivation experiment and LL condition in the intrinsic motivation experiment). The figures of the extrinsic motivation experiment were adapted from Herlambang et al. [9]. Creative Commons License (CC BY-NC 4.0).

Overall, the results of the current experiment are similar to the results of the extrinsic motivation experiment in that participants were more motivated in high motivation blocks (see Fig 8). In both studies, the feeling of fatigue increases over time, which shows that participants did feel fatigue over time regardless of condition. Moreover, participants invested more cognitive effort when the condition was more motivating, which was reflected by similarities in the MF power of HRV in both studies. Cognitive effort seems to be the mediator of the relationship between motivation and performance [23, 65]: Highly-motivated people invest more effort, and this maintains performance. In addition, we found the same effect of exploitation and exploration in the pupil size as other authors have [11]. In the first phase of both experiments, participants learned and tried to do the tasks well (exploitation) that manifested in smaller pupil size over time. Later, the pupil dilated more, searching for more rewarding activities (exploration). Furthermore, participants were more susceptible to distractions when they were not motivated, which is also in line with motivation theory [10].

In summary, the results suggest that intrinsic motivation, as with extrinsic motivation, is essential for explaining the effects of mental fatigue on performance. We propose that intrinsic motivation and extrinsic motivation in both studies share the same process. As time progresses, individuals will invest more effort and exert more control in performing a task that they like (e.g., playing a game, solving a puzzle, working on a hobby–intrinsic motivation) and/or is favorable (it offers more rewards than costs–extrinsic motivation) compared to tasks for which they lack motivation.

Therefore, motivation, both extrinsic and intrinsic, is an important factor in mental fatigue: Even though participants reported becoming fatigued over time, motivation helped participants maintain performance levels and stay engaged with the task by investing more mental effort.

Although this study shows important results, it was limited to a laboratory environment. Therefore, for future research, it is beneficial to conduct a study with real-life tasks that involves vigorous motivation, such as online gameplay [34]. Moreover, a subjective measure of mental states such as the Dundee Stress State Questionnaire (DSSQ) can be used [66]. In addition, it is favorable to have a control group to check a clear comparison between instrinsically motivational condition and nonmotivational one. As an alternative solution to accommodate practice effects, the usage of simple tasks in mental fatigue studies is beneficial [67]. Also, it would be interesting to investigate the effects of relief: In both experiments, we noticed that pupil diameter increased again in the last blocks. We assume that this is caused by participants’ expectation that the experiment would end soon. By including these factors, a more robust theory of mental fatigue and motivation can be developed.

## References

[1] Gergelyfi M, Jacob B, Olivier E, Zénon A. Dissociation between mental fatigue and motivational state during prolonged mental activity. Front Behav Neurosci. 2015; 9:176 10.3389/fnbeh.2015.00176 26217203 PMC4499755

[2] Marcora SM, Staiano W, Manning V. Mental fatigue impairs physical performance in humans. Journal of Applied Physiology. 2009; 106:857–864. 10.1152/japplphysiol.91324.2008 19131473

[3] van der Linden D, Frese M, Meijman TF. Mental fatigue and the control of cognitive processes: Effects on perseveration and planning. Acta Psychologica. 2003; 113:45–65. 10.1016/s0001-6918(02)00150-6 12679043

[4] Helton WS, Russell PN. Rest is best: The role of rest and task interruptions on vigilance. Cognition. 2015; 134: 165–173. 10.1016/j.cognition.2014.10.001 25460389

[5] Helton WS, Russell PN. Rest is still best: The role of qualitative and quantitative load of interruptions on vigilance. Human Factors. 2017; 59(1):91–100. 10.1177/0018720816683509 28146674

[6] Lorist MM, Klein M, Nieuwenhuis S, De Jong R, Mulder G, Meijman TF. Mental fatigue & task control: Planning & preparation. Psychophysiology. 2000; 37:614–625. 10.1017/S004857720099005X 11037038

[7] Warm JS, Parasuraman R, Matthews G. Vigilance requires hard mental work and is stressful. Human Factors. 2008; 50:433–441. 10.1518/001872008X312152 18689050

[8] Boksem MAS, Tops M. Mental fatigue: Cost and benefits. Brain Research Reviews. 2008; 59:125–139. 10.1016/j.brainresrev.2008.07.001 18652844

[9] Herlambang MB, Taatgen NA, Cnossen F. The role of motivation as a factor in mental fatigue Human Factors. 2019; 61:1171–1185. Sage Publishing 10.1177/0018720819828569 30817228 PMC6764012

[10] Hockey GRJ. A motivational control theory of cognitive fatigue In: Ackerman PL, editors. Cognitive Fatigue: Multidisciplinary Perspectives on Current Research and Future Applications. Washington, DC: American Psychological Association; 2011 pp. 167–188.

[11] Hopstaken JF, van der Linden D, Bakker AB, Kompier MAJ. The window of my eyes: Task disengagement and mental fatigue covary with pupil dynamics. Biological Psychology, 2015; 110:100–106. 10.1016/j.biopsycho.2015.06.013 26196899

[12] Brown DMY, Graham JD, Innes KI, Harris S, Flemington A, Bray SR. Effects of prior cognitive exertion on physical performance: A systematic review and meta-analysis. Sports Medicine. 2020; 50:497–529. 10.1007/s40279-019-01204-8 31873926

[13] van Cutsem J, Marcora S, De Pauw K, Bailey S, Meeusen R, Roelands B. The effects of mental fatigue on physical performance: a systematic review. Sports Medicine. 2017; 47:1569–1588. 10.1007/s40279-016-0672-0 28044281

[14] Mizuno K, Tanaka M, Yamaguti K, Kajimoto O, Kuratsune H, Watanabe Y. Mental fatigue caused by prolonged cognitive load associated with sympathetic hyperactivity. Behavioral and Brain Functions. 2011; 7 10.1186/1744-9081-7-17 21605411 PMC3113724

[15] Ting PH, Hwang JR, Doong JL, Jeng MC. Driver fatigue and highway driving: a simulator study. Physiology & Behavior. 2008; 94:448–453. 10.1016/j.physbeh.2008.02.015 18402991

[16] van der Linden D. The urge to stop: The cognitive and biological nature of acute mental fatigue, In: Ackerman PL, editor. Cognitive fatigue: Multidisciplinary Perspectives on Current Research and Future Applications. Washington, DC: American Psychological Association; 2011 pp. 149–164.

[17] Helton WS, Hollander TD, Warm JS, Tripp LD, Parsons K, Matthews G, et al The abbreviated vigilance task and cerebral hemodynamics. Journal of Clinical and Experimental Neuropsychology. 2007; 29:545–552, 10.1080/13803390600814757 17564919

[18] Craig CM, Klein MI. The abbreviated vigilance task and its attentional contributors. Human Factors. 2019; 61 10.1177/0018720818822350 30682267

[19] Earle F, Hockey B, Earle K, Clough P. Separating the effects of task load and task motivation on the effort fatigue relationship. Motivation and Emotion. 2015; 39:467–476. 10.1007/s11031-015-9481-2

[20] Müller T, Apps MAJ. Motivational fatigue: A neurocognitive framework for the impact of effortful exertion on subsequent motivation. Neuropsychologia. 2019; 123:141–151. 10.1016/j.neuropsychologia.2018.04.030 29738794

[21] Boksem MAS, Meijman TF, Lorist MM. Mental fatigue, motivation and action monitoring. Biological Psychology. 2006; 72:123–132. 10.1016/j.biopsycho.2005.08.007 16288951

[22] Rheinberg F, Vollmeyer R. Motivation. 9th ed. Stuttgart: Kohlhammer; 2018.

[23] Westbrook A, Braver TS. Cognitive effort: A neuroeconomic approach. Cognitive Affective & Behavioural Neuroscience. 2015; 15:395–415. 10.3758/s13415-015-0334-y 25673005 PMC4445645

[24] Kurzban R, Duckworth A, Kable JW, Myers J. An opportunity cost model of subjective effort and task performance. Behavioral and Brain Sciences. 2013; 36:661–679. 10.1017/S0140525X12003196 24304775 PMC3856320

[25] Gendolla GHE, Wright R, Richter M. Effort intensity: Some insights from the cardiovascular system In: Ryan R, editor. The Oxford handbook of human motivation. Oxford, UK: Oxford University Press; 2012 pp. 420–438.

[26] Wright RA, Mlynski C, Carbajal I. Outsiders’ thoughts on generating self-regulatory-depletion (fatigue) effects in limited-resource experiments. Perspectives on Psychological Science. 2019; 14(3):469–480, 10.1177/1745691618815654 30925105

[27] Brehm JW, Self EA. The intensity of motivation. Annual Review of Psychology. 1989; 40:109–131. 10.1146/annurev.ps.40.020189.000545 2648973

[28] Deci EL, Ryan RM. Self-determination theory: 93A macrotheory of human motivation, development, and health. Canadian Psychology, 2008; 49:182–185. 10.1037/a0012801

[29] Di Domenico SI, Ryan RM. The emerging neuroscience of intrinsic motivation: A new frontier in self-determination research. Frontiers in Human Neuroscience. 2017; 11 10.3389/fnhum.2017.00145 28392765 PMC5364176

[30] Ryan RM, Deci WL. Intrinsic and extrinsic motivation: Classic definitions and new directions. Contemporary Educational Psychology. 2000; 25:54–67. 10.1006/ceps.1999.1020 10620381

[31] DeCharms R. Personal causation: The internal affective determinants of behavior New York: Academic Press; 1968.

[32] Locke EA, Schattke K. Intrinsic and extrinsic motivation: Time for expansion and clarification. Motivation Science. 2018; 5:277–290. 10.1037/mot0000116

[33] Ryan RM, Moller AC. Competence as central, but not sufficient, for high-quality motivation: a self-determination theory perspective In: Elliot AJ, Dweck CS, Yeager DS, editors. Handbook of Competence and Motivation: Theory and Application. 2nd ed. New York, NY: The Guilford Press; 2017, pp. 214–231.

[34] Hainey T, Connolly T, Stansfield M, Boyle E. The differences in motivations of online game players and offline game players: A combined analysis of three studies at higher education level. Computers & Education. 2011; 57:2197–2211. 10.1016/j.compedu.2011.06.001

[35] Faul F, Erdfelder E, Lang A-G, Buchner A. G*Power 3: A flexible statistical power analysis program for the social, behavioral, and biomedical sciences. Behavior Research Methods. 2007; 39: 175–191. 10.3758/bf03193146 17695343

[36] Palatini P. Need for a revision of the normal limits of resting heart rate. Hypertension. 1999; 33:622–625. 10.1161/01.hyp.33.2.622 10024317

[37] Kunkels YK, van Roon AM, Wichers M, Riese H [Internet]. Cross-instrument Feasibility, Validity, and Reproducibility of Wireless Heart Rate monitors: Novel opportunities for extended daily life monitoring—[cited 2020 Oct 1]. Available from: https://osf.io/4x2ts/10.1111/psyp.13898 PMC10138748 34286857

[38] Lindhardt CL. Report from the Cortrium3 test University Hospital Zeeland, Oncology Department and palliative Units, Naestved: 2016–2017. 2018. 47 p.

[39] Mathôt S, Schreij D, Theeuwes J. OpenSesame: An open-source, graphical experiment builder for the social sciences. Behavior Research Methods. 2012; 44:314–324. 10.3758/s13428-011-0168-7 22083660 PMC3356517

[40] Dalmaijer ES, Mathôt S, van der Stigchel S. PyGaze: An open-source, cross-platform toolbox for minimal-effort programming of eyetracking experiments. Behavior Research Methods. 2014; 46:913–921. 10.3758/s13428-013-0422-2 24258321

[41] Norvig P [Internet]. Solving every sudoku puzzle. [cited 2018 March 8]. Available from: https://norvig.com/sudoku.html

[42] Zijlstra F, van Doorn L. The construction of a scale to measure subjective effort (Technical report) Delft, the Netherlands: Delft University of Technology; 1985.

[43] Galy E, Paxion J, Berthelon C. Measuring mental workload with the NASA-TLX needs to examine each dimension rather than relying on the global score: an example with driving. Ergonomics. 2017; 61 10.1080/00140139.2017.1369583 28817353

[44] Hart SG, Staveland LE. Development of NASA-TLX (Task Load Index): Results of empirical and theoretical research In: Hancock PA, Meshkati N, editors. Human Mental Workload. Oxford, England: North-Holland; 1988 pp. 139–183.

[45] Hill SG, Iavecchia HP, Byers JC, Bittner AC, Zaklade AL, Christ RE. Comparison of Four Subjective Workload Rating Scales. Human Factors. 1992; 34(4):429–439. 10.1177/001872089203400405

[46] Longo L. On the reliability, validity and sensitivity of three mental workload assessment techniques for the evaluation of instructional designs: a case study in a third-level course. In: McLaren BM, Reilly R, Zvacek S, Uhomoibhi J, editors. Proceedings of the 10th International Conference on Computer Supported Education; 2018 March 15–17; Funchal, Madeira. Portugal. CSEDU; 2018. pp. 166–178.

[47] Acharya UR, Joseph KP, Kannathal N, Lim CM, Suri JS. Heart rate variability: a review. Medical and Biological Engineering and Computing. 2006; 44:1031–1051. 10.1007/s11517-006-0119-0 17111118

[48] Berntson GG, Bigger JT, Eckberg DL, Grossman P, Kaufmann PG, Malik M, et al Heart rate variability: Origins, methods, and interpretive caveats. Psychophysiology. 1997; 34:623–648. 10.1111/j.1469-8986.1997.tb02140.x 9401419

[49] Evans S, Seidman L, Tsao J, Lung, Zeltzer L, Naliboff B. Heart rate variability as a biomarker for autonomic nervous system response differences between children with chronic pain and healthy control children. Journal of Pain Research. 2013; 6:449–457. 10.2147/JPR.S43849 23788839 PMC3684221

[50] Kang JH, Kim JK, Hong SH, Lee CH, Choi BY. Heart rate variability for quantification of autonomic dysfunction in fibromyalgia. Annals of Rehabilitation Medicine. 2016; 40(2):301–309. 10.5535/arm.2016.40.2.301 27152281 PMC4855125

[51] Aasman J, Mulder G, Mulder LJM. Operator effort and the measurement of heart-rate variability. Human Factors. 1987; 29:161–170. 10.1177/001872088702900204 3610181

[52] Mulder G, Mulder LJM. Information processing and cardiovascular control. Psychophysiology. 1981; 18:392–402. 10.1111/j.1469-8986.1981.tb02470.x 7267921

[53] Mukherjee S, Yadav R, Yung I, Zajdel DP, Oken BS. Sensitivity to mental effort and test-retest reliability of heart rate variability measures in healthy seniors. Clinical Neurophysiology. 2011; 122:2059–2066. 10.1016/j.clinph.2011.02.032 21459665 PMC3132243

[54] Mulder B, Hofstetter H, van Roon AM. Carspan for windows user’s manual (Version 0.0.1.36) Groningen, the Netherlands: Author; 2009.

[55] Barthelme S. Eyelinker: Load raw data from EyeLink eye trackers. R package version 0.1. Version 0.1 [software]. 2016 [cited 2018 March 8]. Available from: https://cran.r-project.org/web/packages/eyelinker/index.html

[56] Karatekin C. Development of attentional allocation in the dual task paradigm. International Journal of Psychophysiology. 2004; 52:7–21. 10.1016/j.ijpsycho.2003.12.002 15003369

[57] Martins R, Carvalho JM. Eye blinking as an indicator of fatigue and mental load—A systematic review In: Arezes PM, Baptista JS, Barroso MP, editors. Occupational safety and hygiene III. London, UK: Taylor & Francis; 2015 pp. 231–235.

[58] Hockey R. The Psychology of Fatigue: Work, Effort and Control. Cambridge:Cambridge University Press; 2013.

[59] Yang SN. Effects of gaze-contingent text changes on fixation duration in reading. Vision Research. 2009; 49:2843–2855. 10.1016/j.visres.2009.08.023 19715715

[60] Bates D, Mächler M, Bolker B, Walker S. Fitting linear mixed-effects models using lme4. Journal of Statistical Software. 2015; 67:1–48. 10.18637/jss.v067.i01

[61] Fox J, Weisberg S. An R companion to applied regression (2nd ed.). Thousand Oaks, CA: Sage Publications; 2011.

[62] Matthews G, De Winter J, Hancock PA. What do subjective workload scales really measure? Operational and representational solutions to divergence of workload measures. Theoritical Issue in Ergonomics Science. 2019; 21 10.1080/1463922X.2018.1547459

[63] Veltman JA, Gaillard AWK. Physiological indices of workload in a simulated flight task. Biological Psychology. 1996; 42:323–342. 10.1016/0301-0511(95)05165-1 8652751

[64] Steinborn MB, Huestegge L. A walk down the lane gives wings to your brain: Restorative benefits of rest breaks on cognition and self-control. Applied Cognitive Psychology. 2016; 30:795–805. 10.1002/acp.3255

[65] Goodman S, Jaffer T, Keresztesi M, Mamdani F, Mokgatle D, Musariri M, et al An Investigation of the Relationship between Students' Motivation and Academic Performance as Mediated by Effort. South African Journal of Psychology. 2011; 41 10.1177/008124631104100311

[66] Matthews G, Campbell SE, Falconer S, et al Fundamental dimensions of subjective state in performance settings: Task engagement, distress, and worry. Emotion. 2002;:315–340. 10.1037/1528-3542.2.4.315 12899368

[67] Langner R, Steinborn MB, Chatterjee A, Sturm W, Willmes K. Mental fatigue and temporal preparation in simple reaction-time performance. Acta Psychologica. 2010; 133:64–72. 10.1016/j.actpsy.2009.10.001 19878913

